# Reconstructing Grazer Assemblages for Protected Area Restoration

**DOI:** 10.1371/journal.pone.0090900

**Published:** 2014-03-06

**Authors:** Jan A. Venter, Herbert H. T. Prins, David A. Balfour, Rob Slotow

**Affiliations:** 1 School of Life Sciences, University of Kwazulu-Natal, Westville Campus, Durban, South Africa; 2 Department of Biodiversity Conservation, Eastern Cape Parks and Tourism Agency, Southernwood, East London, South Africa; 3 Resource Ecology Group, Wageningen University, Wageningen, The Netherlands; Bangor University, United Kingdom

## Abstract

Protected area management agencies often struggle to reliably reconstruct grazer assemblages due to a lack of historical distribution data for their regions. Wrong predictions of grazing assemblages could potentially affect biodiversity negatively. The objective of the study was to determine how well grazing herbivores have become established since introduction to the Mkambati Nature Reserve, South Africa, how this was influenced by facilitation and competition, and how indigenous grazer assemblages can best be predicted for effective ecological restoration. Population trends of several grazing species were investigated in in order to determine how well they have become established since introduction. Five different conceivable grazing assemblages reflecting a range of approaches that are commonly encountered during conservation planning and management decision making were assessed. Species packing was used to predict whether facilitation, competition or co-existence were more likely to occur, and the species packing of the different assemblages were assessed using ANCOVA. Reconstructing a species assemblage using biogeographic and biological information provides the opportunity for a grazer assemblage that allows for facilitatory effects, which in turn leads to an ecosystem that is able to maintain its grazer assemblage structure. The strength of this approach lies in the ability to overcome the problem of depauperate grazer assemblages, resulting from a lack of historical data, by using biogeographical and biological processes, to assist in more effectively reconstructing grazer assemblages. Adaptive management of grazer assemblage restoration through reintroduction, using this approach would further mitigate management risks.

## Introduction

There have been alarming declines in large mammal populations in protected areas in Africa in the last three decades, which are mainly attributed to habitat loss as well as to consumptive use [1], [2]. In southern Africa, protected areas have been more successful in maintaining their large mammal populations due to effective conservation management [2], [3]. In many of these protected areas, the management interventions are intended to restore ecological patterns and processes that have been affected by anthropogenic disruption [4]–[6]. A common element of these interventions is to reintroduce ‘suitable’ species to, or remove ‘undesirable’ species from, protected areas [7]–[11].

The reintroduction of indigenous herbivores to an ecosystem, reintroduces natural disturbance and processes that are thought to support or promote the re-establishment of local diversity [12]. A reintroduction is considered to be successful if it results in a self-sustaining population [9]. Reintroductions of large mammals to protected areas have had various levels of success over the last few decades [7]–[9]. Most of the unsuccessful reintroductions are attributed to unsuitable habitat [13], animals being non-indigenous (outside of their historical distribution range) [7], and to behavioural problems of the reintroduced animals [14], [15]. Often, however, these explanations are either tautological, or based on suppositions. Conservation authorities opt to use a precautionary approach when deciding which species to introduce or maintain in protected areas, as non-indigenous species are potentially harmful to habitats in which they did not evolve [16], [17]. A critical aspect of this restoration process is the selection of species that are ‘suitable’. In many instances, the past is used to determine which species are suitable, assuming that indigenous species are the most appropriate to achieve restoration objectives [4], [18], [19]. This piecing together of the past is frequently based on historical mammal distribution data (historical records in diaries, journals and correspondence of early explorers, settlers, hunters, missionaries or naturalists as well as from archaeological records and rock paintings) thus leading to the reconstruction of local historic animal assemblages [5], [18]–[20]. But the process of deciding which species is ‘suitable’ or ‘undesirable’ is not an exact science and is open to criticism [19], [20].

Resource competition and facilitation could have a significant effect on the structure and species-richness of large mammal assemblages [21]–[23]. Allometric relationships between body size and metabolic rate, and body size and gut capacity, predict that larger grazers can survive on lower quality forage but require higher bulk intake diets [24], [25]. Conversely, smaller grazers require higher quality forage, but can cope with lower quantities of it [25]. This suggests that for species within the same guild, the more similar in size the more similar a niche they would occupy [21], [26]. This increases the likelihood of competitive interactions [23], [27], [28], despite this interaction being modified by the type of digestive system of these ungulates because ruminants of larger sizes could directly compete with smaller non-ruminants [29]. Ultimately competitive interactions between species could lead to the extinction of the lesser competitor [21], [22]. When the number of one of the herbivore species decreases, competitive release of other species may occur as the effect of a competing herbivore species’ declines [30]. This competitive release can cascade into lower trophic levels, as the forage species composition shifts in response to changed foraging behaviour of the released herbivore species [31].

Hutchinson’s weight ratio theory predicts that character displacement among sympatric competing species leads to sequences in which each species is twice the mass of the next [32]. The higher the species diversity in an area the closer the species packing will be (i.e., reduced difference in body mass among species) [21], [22], [33], [34]. Closer species packing is expected in complex or highly heterogeneous systems [35] as is the case in African grazing ecosystems [21], [36], [37]. The grazing by larger grazers decreases grass biomass as they are better suited to handle high biomass/low nutrient quality forage [21], [38]–[40]. Furthermore, grazing often increases quality and decreases the stem-leaf ratio thus facilitating food intake [41], [42]. These two processes lead to facilitation for smaller grazers [21], [43], which would maximize production and subsequent utilization of the grass layer [38], [43], [44]. Such facilitation will result in a higher total grazer biomass in an area, and in closer species packing [21], [37], [45].

The linking of these type of ecological patterns and processes to historical distribution data is mentioned by several authors [20], [46], but few examples exist where this was actually done [19], [47]. This would suggest that conservation authorities are not using the full set of available tools when making management decisions for protected area restoration, especially when historical distribution data are lacking. This is a concern, as depauperate herbivore assemblages could have negative implications for biodiversity and associated patterns and processes [48], both of which are goals for protected area conservation management [49].

The aim of this study was to determine how well grazing herbivores established since introduction, how it was influenced by facilitation and competition, and how indigenous grazer assemblages can best be predicted for effective ecological restoration. The objectives of the study were therefore to: (1) investigate the role of facilitation and competition on species persistence for eight grazing species post re-introduction; (2) investigate grazer diversity for the protected area under different conceivable assemblages based on biological principles and/or management practice; (3) assess our results against a separate, established, grazer assemblage; (4) critically evaluate current conservation management policy regarding wildlife reintroductions and removals in protected areas and (5) make recommendations for a future management approach.

### Study Area

Mkambati Nature Reserve is a 77-km^2^ provincial nature reserve situated on the east coast of the Eastern Cape Province, South Africa (31°13′–31°20′S and 29°55′–30°04′E). The reserve was established in 1977, before to which it was communal grazing land. The stated objective for the current management of the reserve is the conservation of Mkambati’s unique biodiversity features [50]. The reserve lies within the Indian Ocean Coastal belt bio-region [51] and Pondoland centre of plant endemism [52], and has a mild sub-tropical climate with relatively high rainfall (1200 mm) and humidity [53], [54]. Soils originates from the Natal Group sandstones and are acidic, dystrophic and sandy [55]. Small forest fragments occur in the reserve, and wetland patches are abundant. Some 80% of the reserve consists of Pondoland–Natal Sandstone Coastal Sourveld Grassland [56]. Fires, ignited mainly by poachers, are frequent, which causes a landscape mosaic with nutrient-rich grass patches within a matrix of older, moribund grassland (Venter *pers.observation*), which are considered to be nutrient poor [57], [58].

A total of 1 344 medium to large herbivores were introduced to Mkambati in 1979 to create a hunting ranch that aimed at an international clientele [53]. Species introduced were blesbok (*Damaliscus pygargus phillipsi*), blue wildebeest (*Connochaetes taurinus*), greater kudu (*Tragelaphus strepsiceros*), impala (*Aepyceros melampus*), springbok (*Antidorcas marsupialis*), gemsbok (*Oryx gazelle*), eland (*Tragelaphus oryx*), red hartebeest (*Alcelaphus buselaphus camaa*), Hartmann’s mountain zebra (*Equus zebra hartmannae*), plain’s zebra (*Equus burchelli*) and giraffe (*Giraffa camelopardalis*) [53]. The animals originated mainly from the Kwazulu-Natal Province in South Africa, as well as from Namibia [53]. Approximately 30% (427) of the introduced animals died shortly after introduction (Sunday Times, South Africa, 24 August 1980), with the cause being attributed to “stress and starvation” [53]. The hunting venture failed commercially, after which Mkambati’s status was changed to nature reserve [53]. In 2002 a culling program was initiated, initially to reduce animal numbers, but later (2004 onwards) to remove species that were considered to be non-indigenous from the reserve [59]. The removals were based on recommendations derived from historical mammal distribution data [60], [61], which later shaped the development of a large mammal management policy [59]. Up to 2013, there were still no large predators present in Mkambati Nature Reserve.

## Methods

To determine how well grazing herbivores established in Mkambati since introduction population data were collected from various sources in order to establish population fluctuations from 1979 (when introductions took place) to 2010 (when the most recent game census was carried out) [53], [55], [62]–[65]. We have limited our investigation to mammalian species >2 kg in mass that have grass as an important component (>10%) in their diet. Species mass and feeding type data were sourced from literature [21], [66], [67]. Some of the species investigated (e.g., eland and impala), are mixed feeders [68], [69], which allowed for a different kind of niche differentiation (grazer/browser), but the study was simplified by only considering them as grazers, as was done by Prins and Olff, (1998a) and Olff et al., (2002).

Five conceivable assemblages were investigated, and although assemblages one to four are specific to the circumstances of Mkambati, they do reflect a range of approaches that are commonly encountered during conservation planning and management decision making elsewhere (Table 1).

**Table 1:** The five different grazer assemblages used during the study.

Species	Common name	Mass (kg)#	Assemblage ranks					
			1 Introductionassemblage	2 Status quoassemblage	3 Current policyassemblage	4 Bio-geographicalassemblage	5 iSimangalisoassemblage	
*Pronolagus crassicaudatus*	Natal red rock rabbit	2.2	1	1	1	1		
*Lepus saxatilis*	Shrub hare	2.5	2	2	2	2	1	
*Ourebia ourebi*	Oribi	15			3	3	2	
*Redunca fulvorufula*	Mountain reedbuck	29.5				4	3	
*Antidorcas marsupialis*	Springbok	33	3	3				
*Aepyceros melampus*	Impala	51	4	4		5	4	
*Redunca arundinum*	Southern reedbuck	58	5	5	4	6	5	
*Damaliscus pygargus*	Blesbok	64	6	6				
*Phacochoerus africanus*	Warthog	73.5				7	6	
*Damaliscus lunatus*	Tsessebe	131					7	
*Alcelaphus buselaphus*	Red hartebeest	150	7	7	5	8		
*Connochaetes taurinus*	Blue wildebeest	189	8	8		9	8	
*Oryx gazella*	Gemsbok	195	9					
*Kobus ellipsiprymnus*	Waterbuck	201				10	9	
*Equus burchelli*	Plain’s zebra	235	10	9		11	10	
*Equus zebra*	Hartmann’s mountain zebra	262	11					
*Tragelaphus oryx*	Eland	511	12	10	6	12	11	
*Syncerus caffer*	Buffalo	544			7	13	12	
*Ceratotherium simum*	White rhinoceros	1875				14	13	
*Hippopotamus amphibius*	Hippopotamus	1900				15	14	
*Loxodonta africana*	African elephant	3550			8	16	15	

For each assemblage species body weights were ranked with the smallest species ranked one, the next largest species ranked two, etcetera.

# Body weight data (average of both sexes) from Prins & Olff (1998), Gagnon & Chew (2000) and Skinner & Chimimba (2005).

### Assemblage 1– ‘Introduction’

This assemblage was based on the nine grazer species that were introduced to Mkambati in 1979 together with three species already present at that time (Table 1). The assemblage reflects objectives that were understood to be economic (‘consumptive use’) rather than biological (ecological or biogeographic), and implemented at a time when experience with the restoration of African large herbivore assemblages was still limited.

### Assemblage 2– ‘Status Quo’

This assemblage was based on all grazer species that were still present in Mkambati by the year 2010 (Table 1). The assemblage reflects the outcome of the original decision, the subsequent culling (2002) and decision to remove what was considered to be non-indigenous species (2004), and the performance of the remaining species up to 2010.

### Assemblage 3– ‘Current Policy’

This assemblage was based on all grazer species that would be present in Mkambati if the currently approved large mammal management policy [59] were implemented (Table 1). Assemblage 3 was similar to Assemblage 2, but took into account recommendations based only on historical records [60] to modify the assemblage. All species that were considered to be non-indigenous are removed, and additional species that were considered to be indigenous, but which do not occur in 2010, are reintroduced.

### Assemblage 4– ‘Biogeographic’

This assemblage was based on all grazer species that would be present in Mkambati if a biogeographic approach were followed (Table 1). There is good evidence [51], [70], [71] that Mkambati falls within the same biogeographic region as the Kwazulu-Natal and southern Mozambique coast, which is confirmed by recent new empirical evidence [72]. Based on the above evidence, we accumulated historical distribution data for the Indian Ocean coastal belt bioregion [51] in order to produce a comprehensive species list which included all species that were recorded to have occurred within this region in the past [61], [73]–[76].

### Assemblage 5– ‘Isimangaliso’

This assemblage was based on the grazer assemblage present in the coastal sections of the iSimangaliso World Heritage Site [77] (in Kwazulu-Natal Province), which falls within the same biogeographic region as Mkambati, namely the Indian Ocean coastal belt [51](Table 1). iSimangaliso has similar rainfall patterns (1200–1300 mm p.a.) [78] and soil characteristics (nutrient poor and well leached) when compared with Mkambati [56], [79]. The assemblage reflects an external reference point from within the same biogeographical region, with a well-established indigenous grazer assemblage, of which most have persisted naturally.

Species packing was determined to assess the role of facilitation and competition on species persistence for all assemblage’s following the method of Prins and Olff, (1998a) and Olff et al., (2002), in which the natural logarithm of body mass was regressed against rank number, with the smallest species in the assemblage ranked one, the next species ranked two, etc. When the natural logarithm of species body weight is plotted against the rank number, the slope is predicted to be 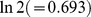 if there is a sequence where each species is exactly twice as heavy as the next [21]. Under such circumstances, the weight ratio 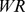 equals 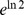 is 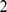. Therefore, the natural logarithm of body weight of the 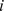-th species 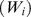 is expected to depend on the rank number 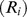 where the regression line follows the function:
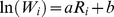
where 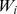 is the body mass of the 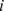-th species in the assemblage and 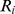 its rank number [21]. The 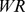 is then obtained by the function



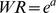



Based on the Hutchinson’s rule, [21] predicted that in a functional group, facilitation is more likely to occur at a weight ratio 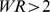 competition at 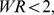 while co-existence will occur at 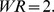 They predicted that when species body mass are too far apart; the larger grazers will keep the grass in a state of utilization in which the vegetation quality is too low for small herbivores, in which case facilitation will not occur. They further predicted that when species are similar in body mass, they might not gain enough from facilitation, and competition will increase [21]. Based on this a weight ratio of 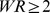 was considered optimal for allowing facilitatory processes needed in an optimal grazer assemblages. Species packing for conceivable assemblages one to four were compared first in order to investigate differences in historical, current and proposed conceivable assemblages within Mkambati.

A one-way analysis of co-variance (ANCOVA) was conducted to determine if there was a significant difference in the degree of species packing for conceivable assemblages one to four. The proposed ‘biogeographic’ assemblage was then compared to an external reference point, i.e. ‘iSimangaliso’, in order to assess accuracy of the predicted grazer assemblage. To determine if there was a difference in species packing for assemblage four and five, a t-test was used. Statistical analysis was conducted using IBM SPSS Statistics for Windows, Version 19.0. (Armonk, NY: IBM Corp.). We compared grazer species abundance among the five different conceivable assemblages according to weight, by generated weight ranges, in which each weight range is more or less half the mass of the next heavier weight range (see [21], [32]). The weight ranges were: mini grazers (2–10 kg), small grazers (11–30 kg), small-medium grazers (31–100 kg), medium grazers (101–200 kg), medium-large grazers (201–500 kg), large grazers (501–1000 kg), mega-grazers (1001–2000 kg) and mega+ -grazers (>2000 kg).

## Results

**Figure 1:**
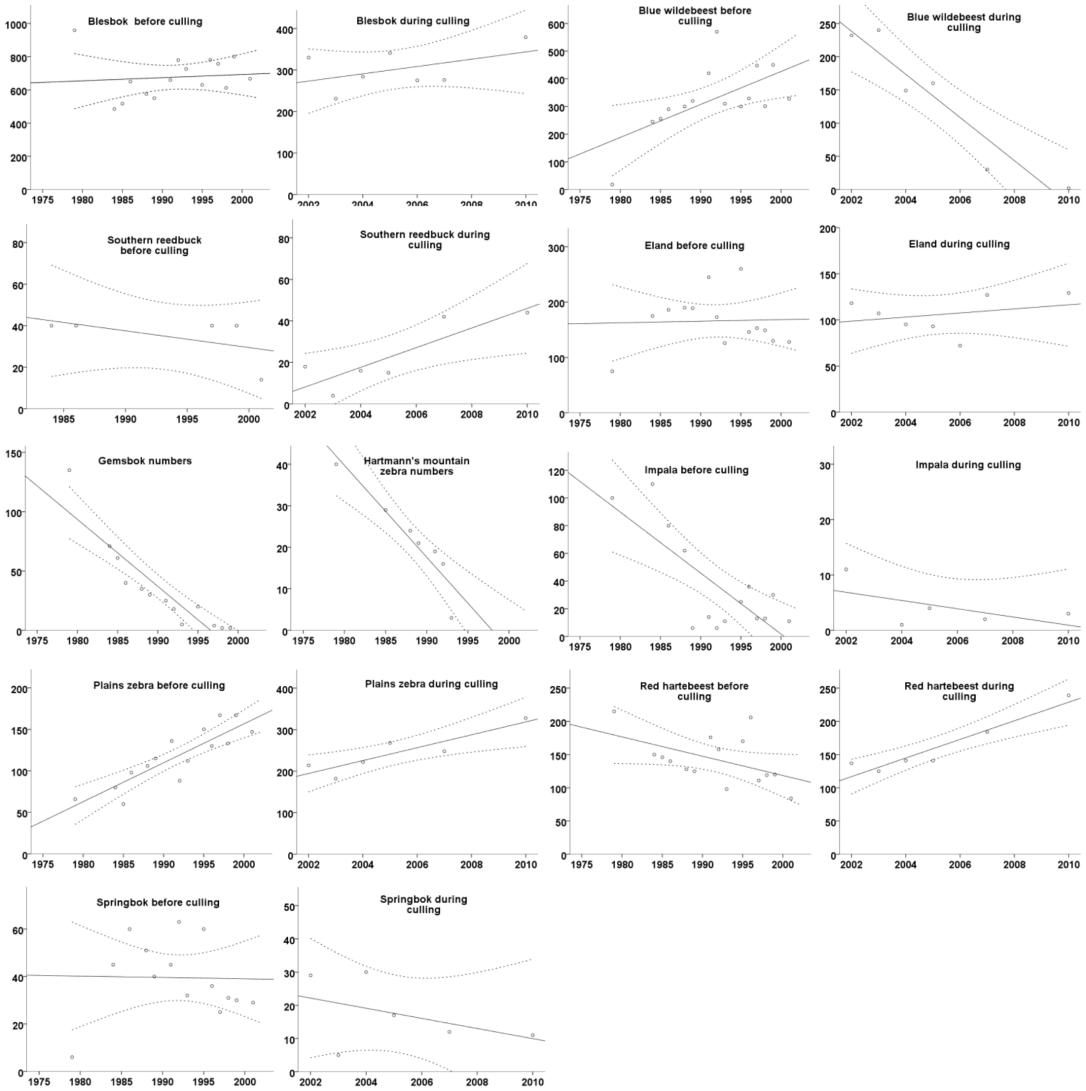
Linear regression lines indication the population growth/decline of red hartebeest, southern reedbuck, eland, blesbok, blue wildebeest, plains zebra, Hartmann’s mountain zebra, gemsbok, impala and springbuck in Mkambati Nature Reserve before and during culling. Dashed lines indicate the 95% CI of the predicted mean.

Dealing with the assumed local indigenous species [60] first, the population of red hartebeest had an initial weak decline 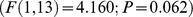 until culling started in 2002, from when population growth showed an upward trend 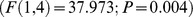 (Figure 1). The number of southern reedbuck remained relatively stable at between 20–50 individuals 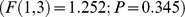 (Figure 1). Numbers of eland fluctuated between 100–200 individuals before and during times when culling took place  (Figure 1).

For the assumed non-indigenous species, numbers of blesbok declined initially after introduction, where-after their numbers fluctuated between 500–800 individuals 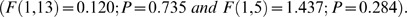 Blue wildebeest showed a strong population growth initially 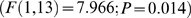 (Figure 1). The population started declining in 2002 due to culling, and was totally removed by 2011 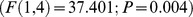 (Figure 1). The numbers of plain’s zebra steadily increased to, and stabilized between 300 and 400 animals by 2010 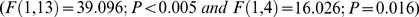 (Figure 1). The number of Hartmann’s mountain zebra started declining after introduction and the species was extinct on Mkambati by 2000, 20 years post-introduction 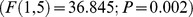 (Figure 1). The numbers of gemsbok declined straight after the introduction until the species went extinct in 1999 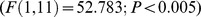 (Figure 1). The population of impala declined after introduction, and crashed to <30 animals 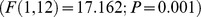 (Figure 1), with only a few (3) being alive in 2010 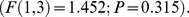 The springbok numbers grew initially until 1992 (±60 individuals) when the population started to decline 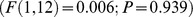 (Figure 1), and by 2012 there were only 11 animals left 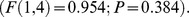 None of the springbok population changes were statistically significant. Of the supposedly indigenous species, some did well after introduction and some less so, and, of the supposedly non-indigenous species, the same can be said (Table 2).

**Table 2:** A summary of the population trends of the large herbivores based on their presumed status of indigenous versus non-indigenous, from when they were introduced to Mkambati Nature Reserve in 1979, until the latest game census in 2010.

Presumed status [60]	Number of species	Increasing population trend	Decreasing population trend	Stable population trend
Indigenous	3	2	0	1
Non-indigenous	7	2	3	2

**Figure 2:**
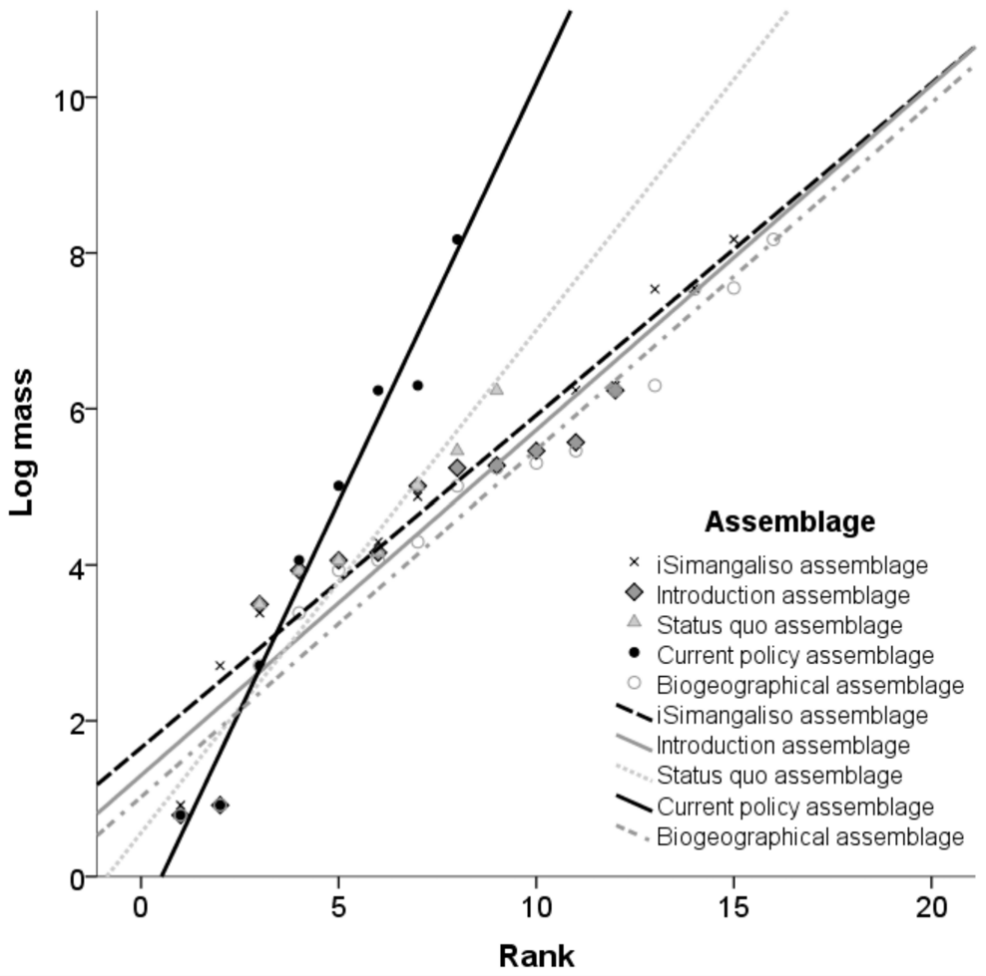
Linear regression lines with the natural logarithm of species’ body mass is plotted against the rank number to indicate the degree of species packing for the ‘Introduction’, ‘Status quo’, ‘Current policy’, ‘Biogeographic’, and ‘iSimangaliso’ grazer assemblages.

When the ANCOVA were performed we first determined that there was a linear relationship between log mass and rank number for each conceivable assemblage, by visually assessing the scatterplot (Figure 2). There was heterogeneity of regression slopes as the interaction term was statistically significant, 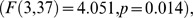 but with visual inspection of the scatterplot it was concluded that this would have a minor effect on the results because the interaction occurred at the very lower end of the scatterplot (Figure 2) see [80]. Standardized residuals for the conceivable assemblages and for the overall model were normally distributed, as assessed by Shapiro-Wilk’s test 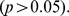 There was homoscedasticity and homogeneity of variances, as assessed by visual inspection of a scatterplot and Levene’s test of homogeneity of variance 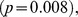 respectively. There were no outliers in the data, as assessed by no cases with standardized residuals greater than ±3 standard deviations. There was a statistically significant difference between the different conceivable assemblages, 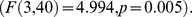 Post hoc pairwise analysis performed with a Bonferroni adjustment indicated a significant difference between the ‘Introduction’ and ‘biogeographical’ assemblages versus the ‘current policy’ assemblage (Table 3). The result of the t-test indicated that there was no significant difference in species packing between the ‘biogeographic’ and ‘iSimangaliso’ assemblages 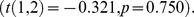 The 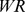 for the ‘status quo’ and ‘current policy’ assemblages were <2, indicating lower species packing and thus higher potential for competitive grazing interactions (Table 4 and Figure 2). The 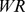 for the ‘introduction’, ‘biogeographical’ and ‘iSimangaliso’ assemblages were >2, indicating higher species packing and thus higher potential for facilitation among grazing species (Table 4 and Figure 2).

**Table 3:** Post-hoc pairwise comparisons indicating the differences between species packing amongst the different conceivable assemblages.

Assemblage	Mean Difference*	Std. Error	Sig.	95% Confidence Interval for Difference	
				Lower Bound	Upper Bound
Introduction assemblage versus Status quo assemblage	−0.371	0.382	1.000	−1.433	0.691
Introduction assemblage versus Current policy assemblage	−1.116	0.398	**0.047**	−2.222	−0.010
Introduction assemblage versus Biogeographical assemblage	0.393	0.336	1.000	−0.539	1.324
Status quo assemblage versus Current policy assemblage	−0.745	0.418	0.493	−1.904	0.415
Status quo assemblage versus Biogeographical assemblage	0.764	0.379	0.303	−0.288	1.815
Current policy assemblage versus Biogeographical assemblage	1.509	0.398	**0.003**	0.404	2.614

*A negative value indicates that the first assemblage have a higher species packing than the second.

**Table 4:** The degree of species packing for the different conceivable assemblages in Mkambati Nature Reserve.

Assemblage	Number of species	R^2^-value	Weight ratio (*WR*)
‘Introduction’	12	0.837	3.669
‘Status quo’	10	0.895	1.751
‘Current policy’	8	0.975	1.751
‘Biogeographic’	16	0.952	2.773
‘iSimangaliso’	15	0.949	5.207

**Figure 3:**
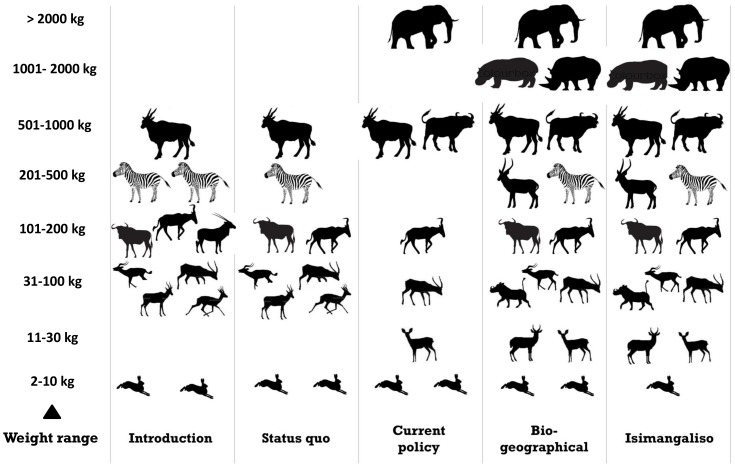
The weight ranges for the grazing species under the five different conceivable assemblages investigated during the study. Weight ranges were grouped as mini grazers (2–10 kg), small grazers (11–30 kg), small-medium grazers (31–100 kg), medium grazers (101–200 kg), medium-large grazers (201–500 kg), large grazers (501–1000 kg), mega grazers (1001–2000 kg) and mega+ grazers (>2000 kg). Conceivable assemblages ‘biogeographic’ and ‘iSimangaliso’ are considered best. Each species is represented by a silhouette.

In order to assess the different species’ ability to persist post introduction we needed to compare ‘introduction’ assemblage with the ‘status quo’ assemblage. The number of species within the small grazer, mega grazer and mega+ grazer body weight ranges, were depauperate in both ‘introduction’ and the ‘status quo’ assemblages (Figure 3). There was a decrease in the number of species in the medium (−2) and medium-large (−1) grazer weight ranges in the period between 1979 and 2010 (i.e., time period between ‘Introduction’ and the ‘status quo’ assemblages)(Figure 3).

There were no species present in the medium-large and mega grazer weight ranges for the ‘current policy’ assemblage (Figure 3). In addition there was only one species per range for the small, small-medium, medium, and mega+ grazer weight ranges (Figure 3). There were between 2 and 3 species for all weight ranges in the ‘biogeographical’ assemblage, except the mega+ weight range, which only had one species (Figure 3). The species packing results for the ‘introduction’, ‘biogeographical’ and ‘iSimagaliso’ assemblages indicate a facilitation assemblage, achievable with a suite of 12; 16 to 15 grazing species, which are relatively evenly spread over all weight ranges. The ‘biogeographical’ and ‘iSimagaliso’ assemblages were similar, except for a depauperate mini grazer weight range in the ‘iSimagaliso’ assemblage (Figure 3).

## Discussion

Forage quality, in many cases, decreases with increasing grass biomass, which imposes an important constraint on net nutrient and energy intake by grazers [21], [22], which is also the case in Mkambati [54], [57]. The presence of larger grazers can decrease grass biomass (because they are better suited to handle high biomass/low nutrient quality forage) [21], [38], [39], and increase quality as well as decrease stem-leaf ratio of forage, thereby facilitating food intake for smaller grazers [21], [41]–[43].

In the case of Mkambati the evidence suggests competitive exclusion resulting in local extinction of some species. This is supported by the species packing values that were <2, as well as evidence of population decline of species in certain weight ranges in the ‘status quo’ assemblage. Shorter term effects that may in addition indicate competitive exclusion can also be seen in the increased population growth of red hartebeest (from 2002 onwards) after the decline of blue wildebeest due to the culling program. Although the ‘introduction’ assemblage showed a facilitation scenario, we reason that it happened in the lower weight ranges, and there was a general lack of facilitation within higher weight ranges, i.e. large and mega grazers upwards. In high rainfall areas (≥750 mm p.a.) mega grazers such as the white rhino and hippopotamus act as influential ecosystem engineers, creating and maintaining short grass swards, which alter habitat for other grazers and change the fire regime [81]–[83]. Elephant, through trampling effect rather than grazing, are probably also able to facilitate availability of grazing resources in dense overgrown areas [44]. This ecosystem engineering role cannot be replicated by smaller grazers [81]. The lack of facilitation effects could thus be linked to the evidence of competition driven species decline in “overpopulated weight ranges” in especially the larger, i.e. medium and medium-to-large weight ranges. It can reasonably be argued, in the case of gemsbok and Hartman’s zebra, which normally occur in more arid areas [84], that poor habitat suitability and their non-indigenous status could have been the main factor responsible for the species demise [7], [13]. This argument could, however, be tautological in that the conclusions are made once the species fails to establish. We argue that, in addition to failure to establish due to a habitat suitability disadvantage, these grazing species may also have been less competitive. Had there been fewer effective competitors and increased facilitation from larger grazers, these species may have been able to overcome the habitat suitability disadvantage and persisted. Our argument, based on missing biological processes, is strengthened by the data showing a prolonged period (20 years) of decline of the said species.

The ‘current policy’ assemblage produced the lowest degree of species packing (lowest 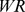), with a resulting increase of likelihood for interspecific competition. In this case, facilitation is unlikely, as there were several gaps in the larger weight ranges (medium-large and mega grazers) of the grazer assemblage. There are two noteworthy observations regarding the ‘current policy’ assemblage. Firstly, a small grazing species assemblage of only eight species in a grass dominated ecosystem is unusual compared to larger species assemblages in other African ecosystems (Mean = 20; ±3 SD; n = 8) [33], [36], [46], [74]. Secondly the lack of ‘mega’ grazers in the assemblage is contrary to the expected assemblage of more abundant mega grazers in high rainfall [85] or high biomass/nutrient poor regions [86]. The ‘current policy’ assemblage, although intended to have a restoration and thus biodiversity conservation objective, may prove to carry the highest risk. In this assemblage, the removal of species might trigger, and could already have triggered, competitive release which may affect lower trophic levels, and cause forage species composition shifts, in response to changed foraging behaviour of the released herbivore species, which could potentially affect biodiversity patterns and processes [31], [48], [87]. The risk to biodiversity could further increase due to a higher fire frequency, caused by fuel load build-up when grass biomass is not effectively cropped by grazers [88]–[90]. This could effectively keep Mkambati in a ‘fire trap’, which currently seems to be the case (Venter, *personal observation*). Furthermore, the lack of larger grazers creates an ecosystem devoid of facilitatory effects which in turn leads to an ecosystem which is unable to maintain its herbivore assemblage structure [21].

The use of only vegetation types in combination with historical distribution data to predict grazer distribution patterns [46], [60] could thus potentially provide inaccurate results [19], [20]. Examples exist where older historical distribution predictions were later proven inaccurate when new evidence was produced [91], [92]. For these reasons, we therefore predict that the current policy approach will not be able to optimally achieve Mkambati’s stated biodiversity conservation purpose [59]. The weakness in this approach lies inherently in the lack of a full grazer assemblage, planned for by using insufficient historical data.

Biogeographic regions are better defined by combining vertebrate data with vegetation data due to a large degree of congruence in distributions caused by the effect of vertebrate distributions [72]. Plant species tend to be responsive to localized environmental conditions, while animal species respond to the broader vegetation structure (i.e. biogeographical regions), which could be a spatially more coherent representation of the floristic patterns [72]. Medium to large grazers in Africa are well known for their ability to move/migrate over large distances, driven by regional seasonal changes in forage conditions [38], [61], [93]–[95], which further supports the use of broader, biogeographical, rather than a narrower vegetation type approach. The ‘biogeographic’ assemblage thus seems to be the more appropriate model to use. This assemblage is similar to an established grazer assemblage in ‘iSimangaliso’ in the same biogeographic region.

The ‘biogeographic’ assemblage, with a full, evenly spread (equal number of species for each weight class) grazer species assemblage, provides the opportunity for a grazing ecosystem that allows for facilitatory effects, that leads to an ecosystem that is able to maintain its herbivore assemblage structure. This in turn maximizes production and utilization in the forage layer which could increase grazer biomass. It would also allow Mkambati to escape from its current ‘fire trap’ of a very high fire return rate. When an assemblage exists where there is a lack of sufficient historical data, the biogeographic approach could be considered to be the more responsible conservation management approach. Furthermore this approach has the highest likelihood of achieving Mkambati’s stated purpose and restoration objectives. The strength of this approach lies in the ability to overcome the problem of depauperate grazer assemblages, caused by a lack of historical data, by using biogeography and ecological processes, to assist in more effectively restoring grazer ecosystems. The proposed approach however, is still very simplistic in nature and various additional factors could be considered. Mouth anatomy and season for example could be important factors that contribute to niche overlap and ecosystem engineering effects [26], [96].

## Management Implications

It remains important that non-indigenous species are not introduced into formal protected areas due to the potential risk associated with such an action [11], [13], [16]. When there is no confirmation from historical data that a species was present in the immediate vicinity of the protected area, but biological or biogeographical patterns contradicts the historical assessment, reintroduction should be planned using a strategic adaptive management approach [97]. This approach should take cognisance of all the potential risks [13], [16] and be focussed on improving incomplete understanding and reducing the identified risks. This should take place through an iterative process of setting reintroduction objectives, implementing reintroduction actions and evaluating the implications of their outcomes for future management action [97]–[99]. This could involve re-introducing certain species (as identified through biogeographical and biological assessment tools), setting thresholds of potential concern (TPC’s) [100], intensively monitor the species’ effect on the ecosystem and the grazer assemblage, later deciding to remove or maintain them, depending on conclusions derived from set TPC’s. A protected area restoration strategy that aims to simulate the natural processes and heterogeneity of a system should thus make full use of all the tools available to reconstruct past species assemblages. These tools are not limited to historical distribution data but include biogeographic and biological approaches. The model proposed in this study should not be seen as the ultimate solution for predicting large herbivore assemblages but rather as a contribution for the development of more scientifically robust and defendable protected area restoration methodology.

## Conclusion

We conclude that it is the larger grazers missing from the Mkambati grazer suite, thus creating an ecosystem devoid of facilitatory effects exerted by these species, which in turn leads to an ecosystem that cannot maintain its herbivore assemblage structure. If certain species are excluded from the system purely based on assumptions derived from local colonial history and early explorer travel habits, the scientific validity of the assessment of their non-indigenous status should be questioned, especially when biological or biogeographical patterns contradict the historical assessment. The functioning of grazing ecosystems is driven by various patterns and processes, and excluding certain species, weight ranges or guilds, could potentially be just as detrimental as including non-indigenous species.

## Acknowledgments

The University of Kwazulu-Natal and Eastern Cape Parks and Tourism Agency for funding the research. Mkambati Nature Reserve staff, students from University of Kwazulu-Natal and students from Pennsylvania State University, Parks and People program for providing field assistance. Dr. Neil Brown from the Pennsylvania State University for providing editorial comments on the initial draft.
